# Engagement of NKG2D on Bystander Memory CD8 T Cells Promotes Increased Immunopathology following *Leishmania major* Infection

**DOI:** 10.1371/journal.ppat.1003970

**Published:** 2014-02-27

**Authors:** Erika J. Crosby, Michael H. Goldschmidt, E. John Wherry, Phillip Scott

**Affiliations:** 1 Department of Pathobiology, School of Veterinary Medicine, University of Pennsylvania, Philadelphia, Pennsylvania, United States of America; 2 Department of Microbiology, Perelman School of Medicine, University of Pennsylvania, Philadelphia, Pennsylvania, United States of America; National Institute of Health, United States of America

## Abstract

One of the hallmarks of adaptive immunity is the development of a long-term pathogen specific memory response. While persistent memory T cells certainly impact the immune response during a secondary challenge, their role in unrelated infections is less clear. To address this issue, we utilized lymphocytic choriomeningitis virus (LCMV) and *Listeria monocytogenes* immune mice to investigate whether bystander memory T cells influence *Leishmania major* infection. Despite similar parasite burdens, LCMV and *Listeria* immune mice exhibited a significant increase in leishmanial lesion size compared to mice infected with *L. major* alone. This increased lesion size was due to a severe inflammatory response, consisting not only of monocytes and neutrophils, but also significantly more CD8 T cells. Many of the CD8 T cells were LCMV specific and expressed gzmB and NKG2D, but unexpectedly expressed very little IFN-γ. Moreover, if CD8 T cells were depleted in LCMV immune mice prior to challenge with *L. major*, the increase in lesion size was lost. Strikingly, treating with NKG2D blocking antibodies abrogated the increased immunopathology observed in LCMV immune mice, showing that NKG2D engagement on LCMV specific memory CD8 T cells was required for the observed phenotype. These results indicate that bystander memory CD8 T cells can participate in an unrelated immune response and induce immunopathology through an NKG2D dependent mechanism without providing increased protection.

## Author Summary

Cutaneous leishmaniasis has a wide spectrum of clinical presentations, from mild self-healing lesions to severe chronic infections. Differences in each individual's response are related to pathogen dose and the genetic and physiological status of the host, but exactly what causes the broad spectrum of disease is not well understood. Here we show that previous infection with a viral or bacterial pathogen led to increased immunopathology associated with *L. major* infection. This increase in immunopathology was not associated with any changes in parasite control and was characterized by an exaggerated inflammatory infiltrate into the site of infection. Ultimately, this increase in immunopathology was dependent on the presence of memory CD8 T cells from the previous infection and their expression of the NK cell receptor NKG2D, as depletion of these cells prior to infection with *L. major* or blockade of this receptor during infection ameliorated the disease. Our work suggests that the immunological history of a patient may be playing an underlying role in the pathology associated with leishmania infection and could be an important consideration for the understanding and treatment of this and other human diseases. This work also identifies the NKG2D pathway as a potential new target for therapeutic intervention.

## Introduction

Over time and with increased immunological experience, our pool of memory CD8 T cells increases, resulting in a large repertoire of memory T cells that are able to protect against previously encountered infectious agents. This protection is thought to be life long and pathogen specific. Less well studied is the ability of these memory T cells to respond in a TCR-independent fashion that might influence the outcome of an unrelated infection. A role for bystander memory T cells (i.e. memory T cells that are activated independent of TCR stimulation) has been described in viral infections, where subsequent heterologous viral challenge leads to reactivation of memory CD8 T cells and increased protection [1]. Similarly, activation of bystander memory CD8 T cells has also been observed in bacterial and parasitic infections, leading to the notion that an accumulation of memory CD8 T cells may promote increased resistance to unrelated infections [2]–[5]. Work from several groups has shown that CD8 T cells have a remarkable ability to become activated by cytokines in a TCR-independent manner, characterized by rapid acquisition of effector functions [6]–[9]. However, while memory CD8 T cells can promote increased resistance, in some situations activation of bystander CD8 T cells may be pathologic and has even been shown to play a role in autoimmune diseases [10]. The inflammatory signals that induce a bystander CD8 T cell to be protective versus pathologic in different disease states is poorly understood.

Cutaneous leishmaniasis has a wide spectrum of clinical presentations, from mild self-healing lesions to severe chronic infections. Control of these parasites is primarily dependent upon the development of a strong CD4 Th1 response, which leads to the production of IFN-γ that activates macrophages and kills the parasites [11], [12]. Under some conditions, CD8 T cells also play a protective role by producing IFN-γ to both directly activate macrophages, and promote the development of a strong CD4 Th1 response [13], [14]. However, disease severity in leishmaniasis is only partially dependent upon the parasite burden, and some forms of the disease are associated with very few parasites but an exaggerated immune response [15]–[17]. The factors that determine the severity of the disease remain poorly defined, but may include decreased expression of IL-10 or the IL-10R, thereby leading to increased production of IFN-γ, TNF-α and/or IL-17 [18]–[22]. Additionally, in some patients there is a strong correlation between the severity of the disease and the number of CD8 T cells within the lesions [23]–[25]. Instead of expressing IFN-γ, however, the majority of these CD8 T cells express granzyme B (gzmB) [24], [25]. Recently, we have shown that these cytolytic CD8 T cells promote pathology, rather than resistance [26]. Thus, while IFN-γ producing CD8 T cells may be protective in leishmaniasis, it appears that gzmB expressing CD8 T cells are associated with enhanced disease.

In this study, we found that bystander CD8 memory T cells exacerbate disease following infection with *L. major*. We infected mice with LCMV or *Listeria* to generate a large pool of memory CD8 T cells, and challenged the mice with *L. major*. Following infection with *L. major*, LCMV or *Listeria* immune mice develop significantly larger lesions than control mice characterized by increased numbers of monocytes, neutrophils, and CD8 T cells but no change in the parasite burden. Depletion of CD8 T cells following LCMV infection, but prior to challenge with *L. major*, resulted in a loss of the observed pathology. Strikingly, when anti-NKG2D blocking antibodies were given, the increased immunopathology in LCMV immune mice was completely abrogated, indicating that engagement of the NKG2D pathway in CD8 T cells was responsible for the observed immunopathology. Thus, this work demonstrates that a bystander memory CD8 T cell population can contribute to the chronic inflammation and immunopathology associated with cutaneous leishmaniasis.

## Results

### LCMV memory T cells migrate into leishmanial lesions and upregulate gzmB

**Figure 1:**
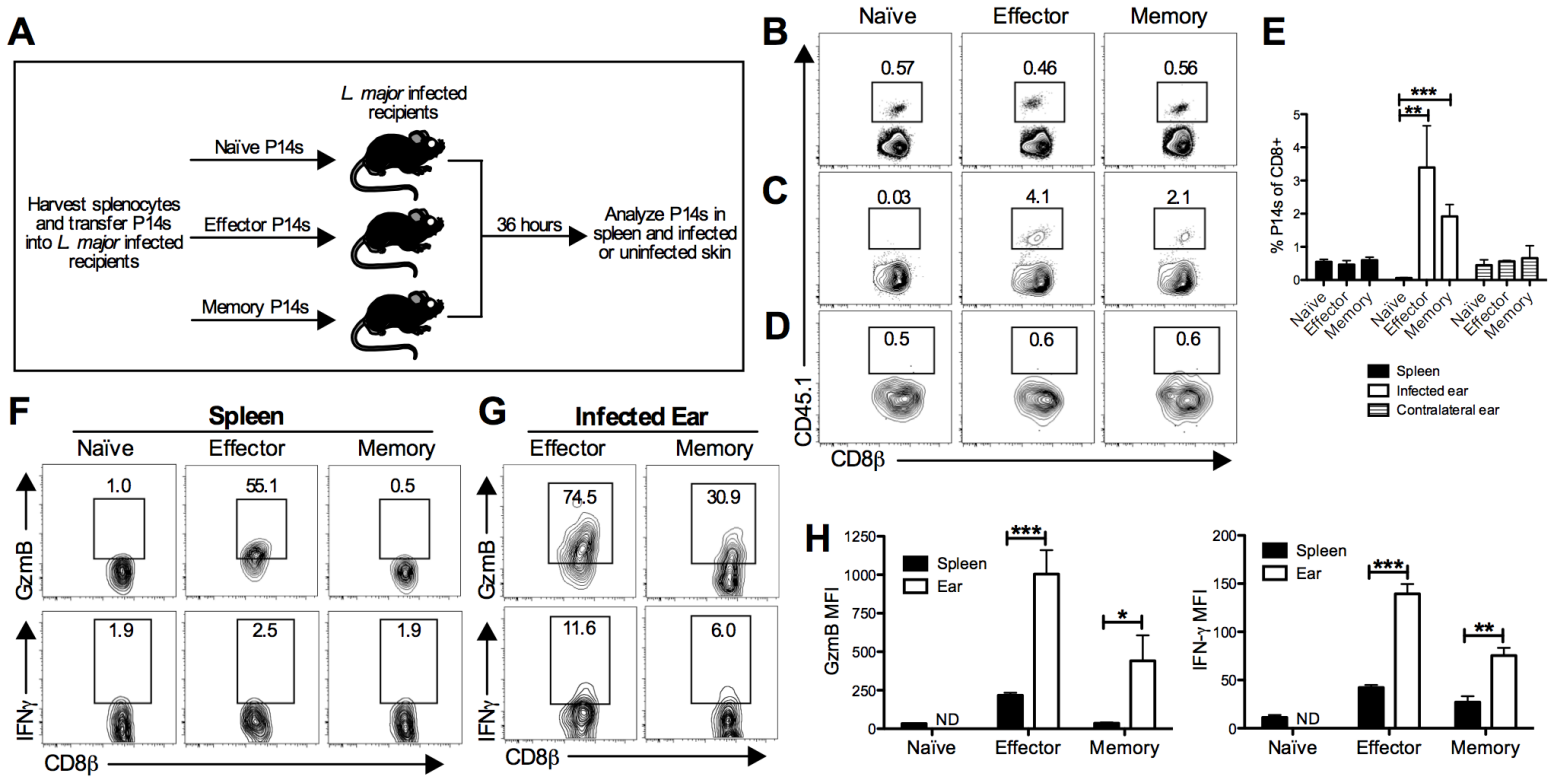
LCMV memory T cells migrate to leishmanial lesions and upregulate gzmB expression. B6 mice received CD45.1+ P14 cells (P14 chimeras) and were infected with LCMV for 5 days (effector time point) or 30 days (memory time point). At the indicated time post LCMV infection, splenocytes were harvested from naïve P14 mice or P14 chimeras and the numbers of P14 CD8 T cells were quantified. Equal numbers (5×10^5^) of P14 CD8 T cells were transferred into congenically marked B6 mice that had been infected with *L. major* for 2 weeks (A). After 36 hours, spleens (B), infected ears (C), and contralateral uninfected ears (D) were harvested from the recipients and P14 frequency was analyzed by flow cytometry (E). P14 CD8 T cells present in the spleen (F) or infected ear (G) were incubated with BFA alone for 5 hours and analyzed for production of gzmB and IFN-γ. The relative mean fluorescence intensity for each was calculated (H). Data are representative of two independent experiments (n = 3–5 per group).

CD8 T cells can be recruited to tissues from the blood independent of antigen specificity, which raises the possibility that they may influence a subsequent infection [27]. To determine if LCMV memory T cells can migrate into *L. major* infected skin, we performed a transfer experiment using transgenic P14 CD8 T cells. These cells express a TCR specific for the LCMV peptide GP33. P14 T cells were transferred into CD45 congenic C57BL/6 (B6) mice, which were then infected with LCMV. The P14 T cells were harvested during the effector phase (day 5 post-infection) or memory phase (day 30 post-infection) of LCMV infection. Naive, effector, or memory P14 T cells were then transferred into three different groups of congenic mice that had been infected with *L. major* for 2 weeks (Figure 1A). The spleen, infected skin, and uninfected control skin were analyzed for the presence of the donor cells 36 hours later. P14 T cells were readily detected in the spleen at similar frequencies in each group (Figure 1B and 1E). As predicted based on previous literature [28], [29], naïve P14 T cells were unable to migrate into *L. major* infected skin (Figure 1C). Similarly, regardless of the activation state of the transferred cells, none were detected in the uninfected control skin (Figure 1D and 1E). However, both effector and memory P14 T cells were found in the *L. major* infected skin (Figure 1C and 1E).

Given that both the effector and memory P14 T cells were able to migrate into the inflamed skin, we wanted to evaluate two of their main effector functions, cytolysis and cytokine production. To assess these functions, cells were harvested and incubated with brefeldin A (BFA) without additional stimulation for 5 hours. We assessed gzmB levels as an indicator of the potential cytotoxic function and, given its critical role in controlling pathogens, we assessed IFN-γ production. As expected, a large percentage of effector P14 cells in the spleen expressed gzmB, whereas naïve and memory P14 T cells did not (Figure 1F and 1H). P14 cells from all three groups were negative for IFN-γ in the spleen (Figure 1F and 1H). In contrast, both effector and memory P14 T cells in the infected ear expressed gzmB and IFN-γ, although the IFN-γ levels were quite low (Figure 1G and 1H). These data suggest that both effector and memory T cells not only migrate into leishmania lesions, but also become activated upon entering the inflamed tissue.

### Previous heterologous infection exacerbates the immunopathology of a subsequent *L. major* infection

**Figure 2:**
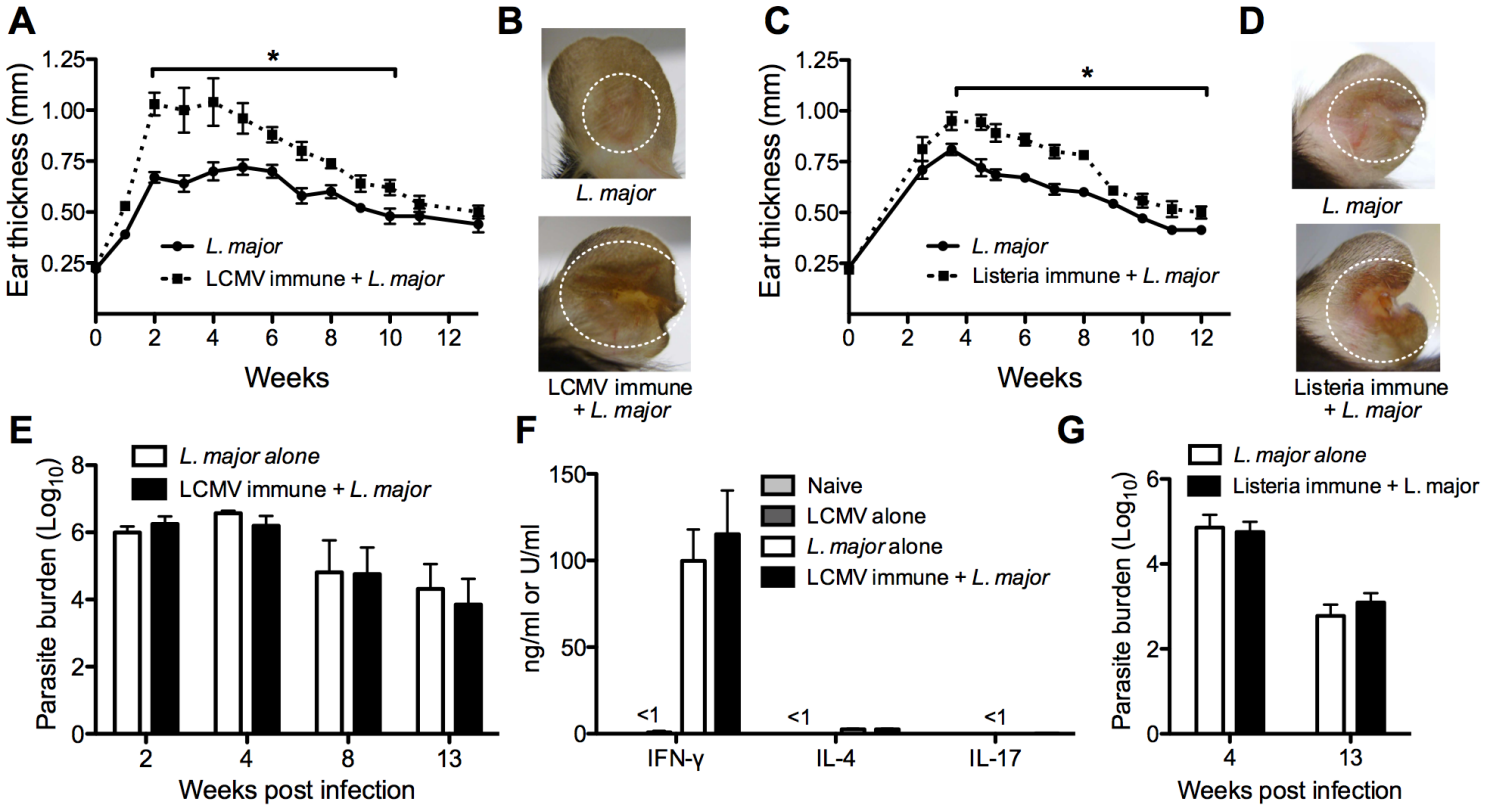
Previous heterologous infection increases leishmanial lesion size with no effect on parasite control. B6 mice were infected with LCMV Armstrong or left uninfected. After 30 days, mice were infected with *L. major* and ear thickness was measured weekly (A). Pictures were taken 4 weeks post *L. major* infection (B). B6 mice were infected with *Listeria*-OVA or left uninfected and 30 days later boosted again with *Listeria*-OVA or left uninfected. Thirty days after the boost, all mice were infected with *L. major* and ear thickness was measure weekly (C). Pictures were taken 5 weeks post *L. major* infection (D). Infected skin from both groups was taken at various time points post infection and parasite burden was assessed using a limiting dilution assay (E and G). At 4 weeks post infection in the LCMV immune mice, draining lymph nodes were removed and cultured with leishmanial antigen or media alone. After 72 hours, supernatants were removed and analyzed for IFN-γ, IL-4, or IL-17 by ELISA (F). Cells cultured with media alone did not produce any cytokines (data not shown). These data are a compilation of five independent experiments (n = 5–10 mice per group per time point; A, B, E, and F) or representative of two independent experiments (n = 10–16 mice; C, D, and G).

Having seen that LCMV memory T cells readily entered the skin in response to leishmania infection, we wanted to determine if previous infection with LCMV affected the course of subsequent *L. major* infection. For this, we infected B6 mice with LCMV or left them uninfected and waited thirty days for the infection to clear and a stable memory T cell population to form. All mice were then infected with *L. major* and lesion development was monitored over time. Given that LCMV induces a strong Th1-type immune response, we predicted that the LCMV immune mice would be more resistant to *L. major* infection. However, LCMV immune mice challenged with *L. major* had significantly larger lesions than did mice that had received *L. major* alone, and the lesions exhibited exacerbated gross pathology (Figure 2A and 2B). In some mice the severe inflammatory response led to permanent loss of tissue. Despite this increase in lesion size, there were no differences in parasite burden within the lesions between the two groups at any time point examined (Figure 2E). In summary, not only were the LCMV immune mice susceptible to infection, the lesions were larger and more severe than mice that received *L. major* alone.

To determine if this was a phenotype specific to LCMV, we did similar experiments with *Listeria monocytogenes*. *Listeria* immune or naïve mice were infected with *L. major* and lesion development was monitored. As with the LCMV immune mice, *Listeria* immune mice had larger lesions with greater pathology than did control mice, with no corresponding change in parasite burden (Figure 2C, 2F, and 2G). Taken together, these unexpected data show that previous heterologous infections can significantly exacerbate the disease associated with *L. major* infection, without any increased ability to control the parasite.

### Leishmania-specific cytokine responses are unaffected by previous infection with LCMV

To determine if a previous LCMV infection impacted the systemic anti-leishmanial response, cells from the draining lymph node were isolated at various times after *L. major* infection from control and LCMV immune mice, and cultured with either media alone or leishmanial antigen. Supernatants were collected at 72 hours and analyzed for the presence of IFN-γ, IL-4 or IL-17 by ELISA (Figure 2F). As expected, cells from *L. major* infected mice produced significant levels of IFN-γ in response to leishmanial antigen. Similar levels of IFN-γ were produced in response to stimulation by cells from LCMV immune *L. major* infected mice, which is consistent with the fact that there were no differences in parasite control between mice that had previously been infected with LCMV and controls. Correspondingly, the levels of IL-4 and IL-17 were quite low in both groups of mice. When lymph node cells from control naïve or LCMV immune mice were cultured with leishmanial antigen, very low levels of IFN-γ and no IL-4 or IL-17 were detected (Figure 2F). Data shown here is from 4 weeks post *L. major* infection, however the same results were also seen at earlier (1–2 weeks) and later (8–12 weeks) time points throughout the infection (data not shown).

### Increased cellular infiltration into the lesions of LCMV immune *L. major* infected mice

**Figure 3:**
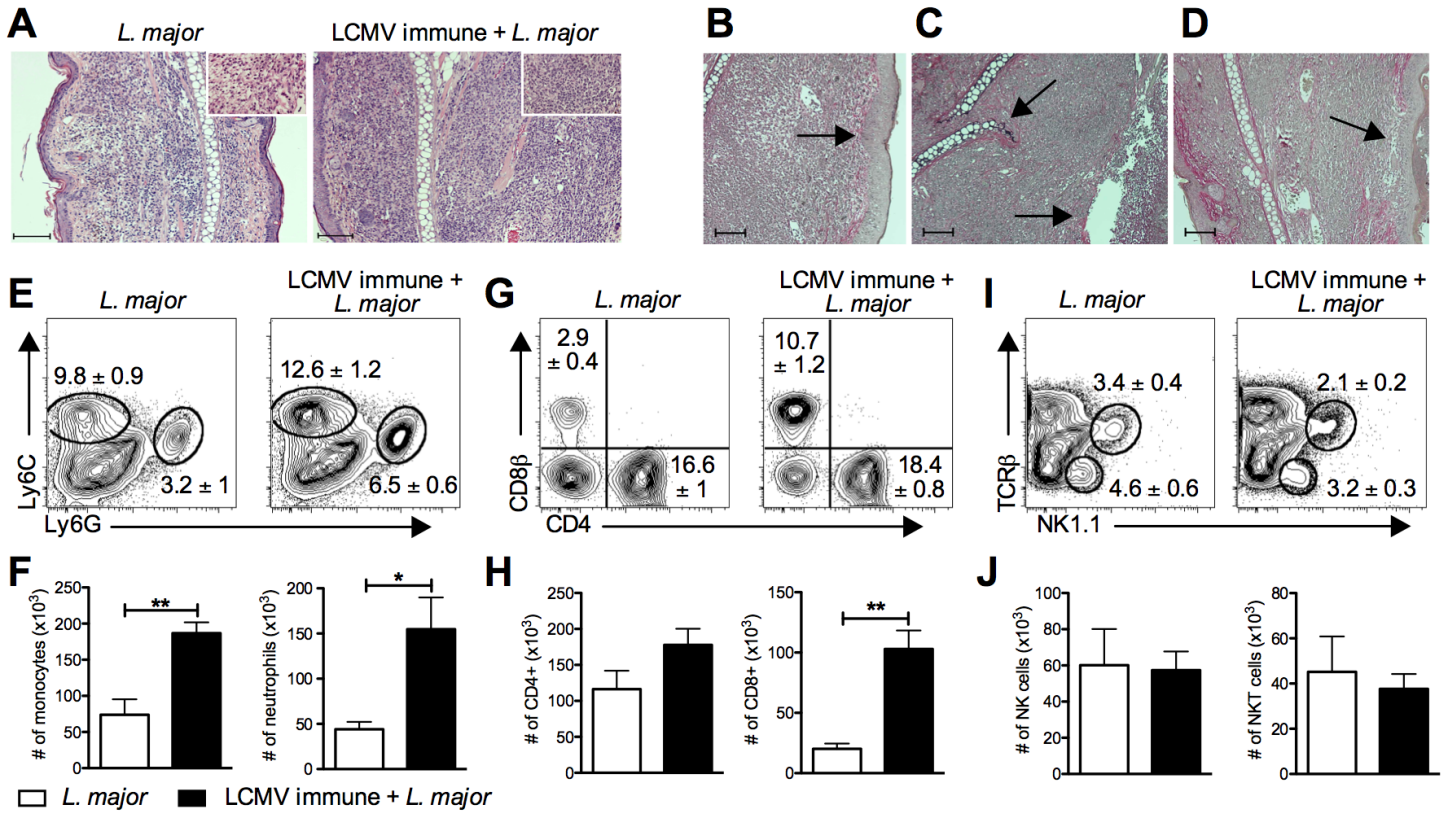
Increased cellular infiltration into the lesion of LCMV immune *L. major* infected mice. B6 mice were infected with LCMV Armstrong or left uninfected. After 30 days, mice were infected with *L. major*. After 4 weeks, infected ears were taken and fixed for histological analysis (A–D) or digested for flow cytometric analysis (E–H). Fixed ear tissue was sectioned and stained with hematoxylin and eosin (H&E) and imaged at 10× magnification or 40× magnification (inlay)(A). A Verhoeff's stain was also done on fixed ear tissue from LCMV immune *L. major* infected mice to stain for collagen and imaged at 10× magnification (B–D). Scale bars represent 100 µm. Damage unique to the LCMV immune lesions is highlighted by arrows, specifically epidermal thickening (B), cartilage destruction (C), epidermal ulceration (C), and loss of dermal collagen (D). Digested ears were stained for surface markers of monocytes and neutrophils (E), T cells (G), or NK cells (I). Results are shown in the form of representative plots (E, G, and I) and total cell numbers (F, H, and J). Samples were pregated on live, CD45+, TCRβ−, CD11b+ events (E) or on live, CD45+ events (G and I). Histological data are representative of two independent experiments (n = 4–5 mice per group; A–D). Flow cytometric data is a representative of five independent experiments (n = 4–5 per group; E–J). Percentages are shown as mean ± SEM.

To understand the nature of the increased pathology seen in LCMV immune mice infected with *L. major*, we examined the cellular infiltration into the lesions. Staining leishmanial lesions in control and LCMV immune mice with hematoxylin and eosin identified an infiltration of leukocytes, predominantly of mononuclear and polymorphonuclear cells. However, the magnitude of the inflammatory response was significantly greater in the lesions from LCMV immune mice (Figure 3A). In addition, to get a better understanding of the structural changes occurring in the lesions from LCMV immune mice, a Verhoeff's stain for extracellular matrix proteins was used. These sections exhibited epidermal thickening (hyperplasia) and spongiosis (Figure 3B), decreased dermal collagen and increased ulceration (Figure 3C and 3D), as well as destruction of the ear cartilage (Figure 3C). To further characterize the cellular infiltrate, we analyzed the cellular composition of the cells in the lesions by flow cytometry. As expected from the histology, we saw a significant increase in the number of both monocytes and neutrophils present in the lesions of LCMV immune *L. major* infected mice compared to controls (Figure 3E and 3F).

Further analysis by flow cytometry revealed that there was also a significant increase in the number of CD8 T cells in the lesions of LCMV immune mice (Figure 3G and 3H). The increase in CD8 T cells was seen as early at 2 weeks post *L. major* infection and was maintained throughout the entire course of infection (data not shown). In contrast, there was no change in the number of CD4 T cells present in the lesion (Figure 3G and 3H). Similar to CD4 T cells, the number of NK and NKT cells was not different in the two groups (Figure 3I and 3J).

To determine if the differences in frequencies of T cells in the lesions reflected differences in the blood, we analyzed T cells circulating in the blood of these animals at the same time post *L. major* infection. While a similar frequency and activation profile of CD4 T cells was observed between the two groups (Figure S1A), there was a significant increase in the percent of CD8 T cells circulating in the blood of LCMV immune *L. major* infected mice relative to control mice. This increase was due to an expansion in the effector memory population of CD8 T cells (Figure S1B) [30]. These results, taken together with our adoptive transfer experiments (Figure 1), are consistent with the idea that LCMV memory CD8 T cells circulating in the blood are recruited into the leishmanial lesions.

### CD8 T cells in leishmanial lesions express gzmB and markers of degranulation

**Figure 4:**
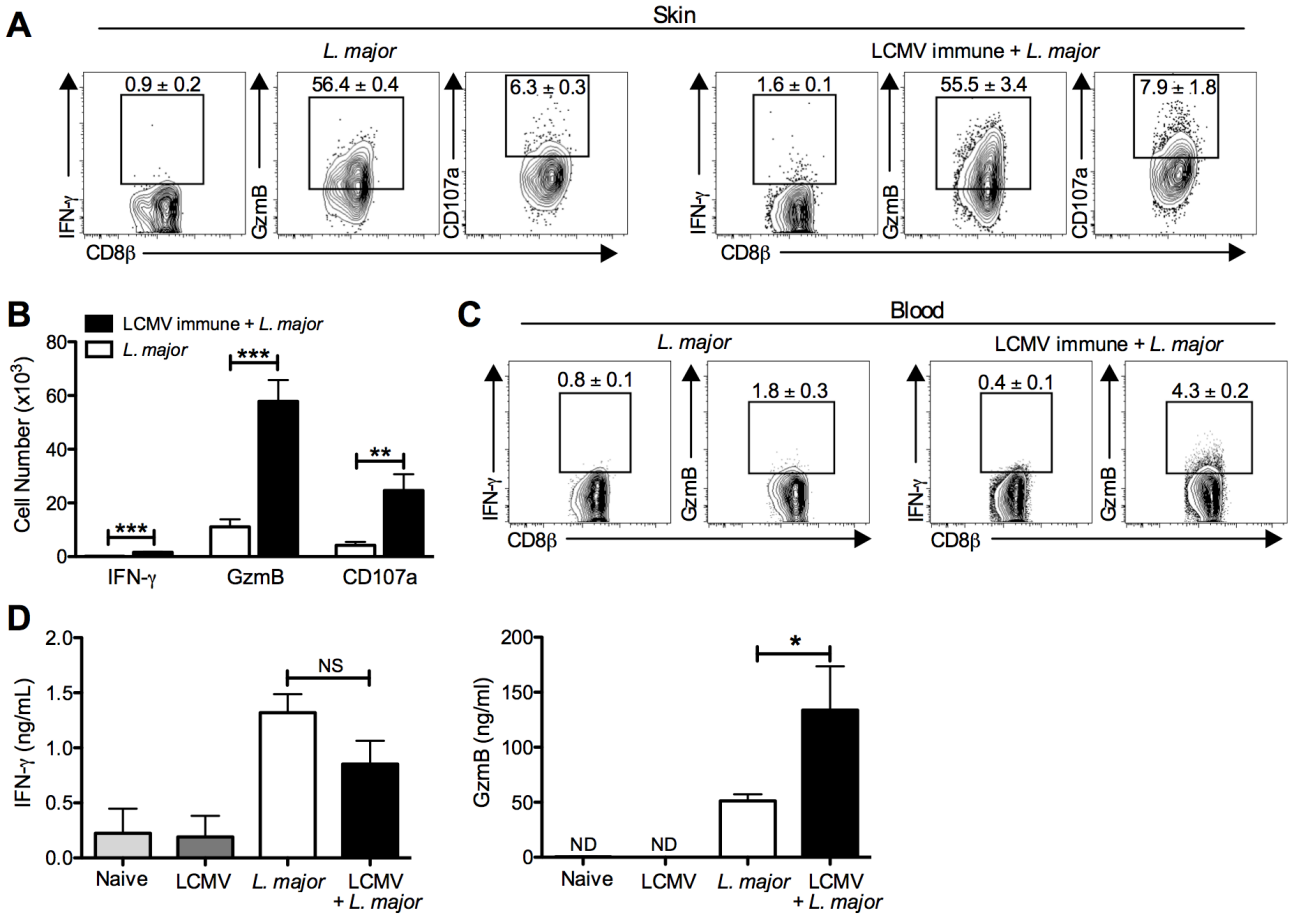
CD8 T cells infiltrating the leishmanial lesions express gzmB but low levels of IFN-γ. B6 mice were infected with LCMV Armstrong or left uninfected. After 30 days, mice were infected with *L. major*. After 4 weeks, infected ears (A) and peripheral blood (C) were taken for analysis by flow cytometry. Cells from the infected ears were incubated with BFA, monensin and CD107a antibody for 5 hours and then stained for additional cell surface markers and intracellular proteins. Representative dot plots (A) and total cell numbers (B) are shown. Peripheral blood was taken and white blood cells were isolated and stained for cell surface markers and intracellular proteins. Representative dot plots are shown (C). Whole ear tissue was homogenized and supernatants were analyzed for gzmB and IFN-γ by ELISA (D). Flow data are representative of five independent experiments (n = 4–5 mice per group). Ear supernatant data are representative of two independent experiments (n = 4 mice per group). Percentages are shown as mean ± SEM.

Having seen a significant increase in the number of CD8 T cells within leishmanial lesions in LCMV immune mice, we next evaluated the function of these CD8 T cells. Cells were taken from the lesions of either control or LCMV immune mice and incubated with BFA for 5 hours. We were particularly interested in examining the production of IFN-γ and gzmB, two of the major effector proteins of CD8 T cells. CD8 T cells from either group made little detectable IFN-γ (Figure 4A), and no detectable levels of TNF-α or IL-17 (data not shown). However, we found that the deficit in IFN-γ production was overcome if CD8 T cells were stimulated with PMA and ionomycin (data not shown), indicating that these cells were capable of making IFN-γ, but might not be receiving a sufficient stimulus to do so in the leishmanial lesions. In contrast to IFN-γ, the majority of CD8 T cells from the lesion express gzmB (Figure 4A). In addition, there were significantly more CD8 T cells in the LCMV immune *L. major* challenged mice expressing gzmB than in mice that received *L. major* alone (Figure 4B). CD8 T cells from the blood at the same time post-infection did not express either of these proteins (Figure 4C), suggesting that upregulation of gzmB was occurring within the lesion, although we cannot rule out the possibility that a small number of gzmB expressing CD8 T cells were preferentially recruited to the lesions.

Given the high expression of the cytotoxic protein gzmB, we wanted to assess whether CD8 T cells from the lesions were actively degranulating. CD107a is expressed in the cytotoxic granules and will be expressed on the surface of actively degranulating cells. To assess CD107a expression as a marker for degranulation, we added CD107a antibody to cultures of cells taken from the lesions that were incubated for 5 hours with BFA and monensin. There was detectable CD107a expression by CD8 T cells from the lesions of both control and LCMV immune mice, indicating that these CD8 T cells were not only expressing gzmB but were also actively degranulating (Figure 4A). As with the gzmB expression, there were significantly more CD107a expressing CD8 T cells in the LCMV immune lesions than in mice that had received *L. major* alone (Figure 4B). Degranulation by NK cells in the lesions as measured by CD107a expression was not different in the two groups (data not shown).

The differential production of IFN-γ and gzmB by CD8 T cells was unexpected, and to directly determine whether the differences we saw by intracellular flow cytometry were reflected in the actual levels of IFN-γ and gzmB present in the lesions, we homogenized the lesions and collected the supernatants for analysis by ELISA. Consistent with the flow cytometry, similar levels of IFN-γ were detected in the lesions of LCMV immune *L. major* infected and control mice (Figure 4D). Given that there were many more CD8 T cells present in the LCMV immune *L. major* infected lesions compared with controls, this result suggests that these CD8 T cells were not producing significant amounts of IFN-γ. In contrast, there was significantly more gzmB in supernatants from lesions taken from LCMV immune *L. major* infected mice compared to controls (Figure 4D). There was no detectable gzmB in supernatants taken from the skin of naïve mice or LCMV immune mice. These results support the flow cytometry data indicating that neither group of CD8 T cells make significant amounts of IFN-γ, while gzmB levels are significantly upregulated in LCMV immune *L. major* infected skin.

Finally, CD4 T cells from the lesions were also analyzed, and while they exhibited detectable levels IFN-γ, there were no differences seen between the two groups (data not shown). This data is consistent with what we saw upon restimulation responses to leishmanial antigen by draining lymph node cells and homogenized ear supernatants (Figures 2F and 4D). Given that CD4 T cell derived IFN-γ is crucial for controlling the parasites, this is also consistent with the data showing that the parasite burden of both groups were not different (Figure 2E).

### LCMV specific CD8 T cells infiltrate leishmanial lesions

**Figure 5:**
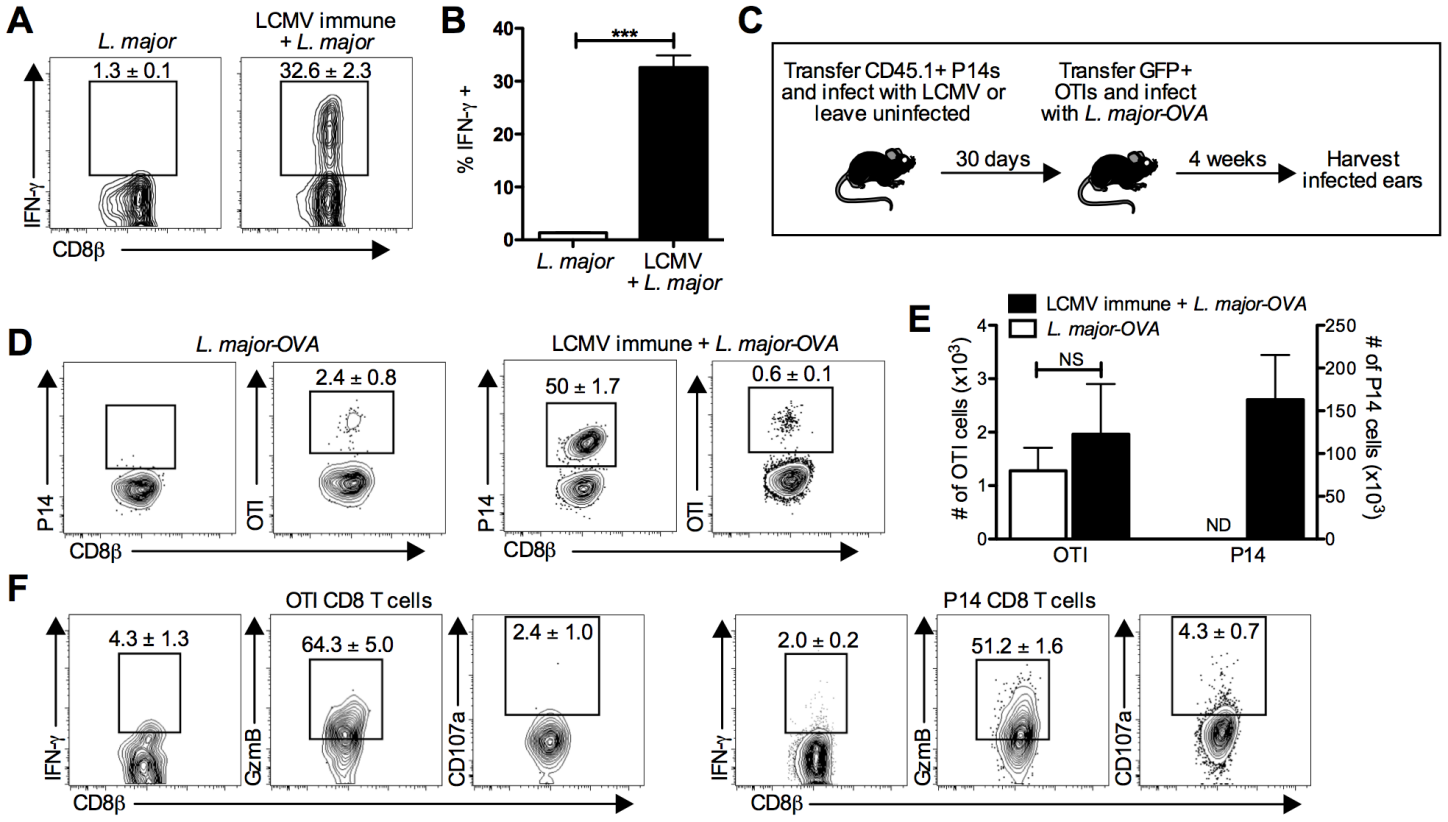
LCMV specific CD8 T cells are present in the leishmanial lesion and express gzmB but low levels of IFN-γ. B6 mice were infected with LCMV Armstrong or left uninfected. After 30 days, mice were infected with *L. major*. After 4 weeks, *L. major* infected skin was taken and incubated with a pool of 20 LCMV derived peptides and BFA for 6 hours before staining for surface and intracellular proteins. Representative plots (A) and pooled data (B) are shown. Data are representative to 2 independent experiments (n = 5 per group). P14 T cells were transferred into B6 mice and the next day a group was infected with LCMV. After 30 days, GFP+ OTI T cells were transferred into all groups and mice were infected with *L. major*-OVA (C). After 4 weeks, *L. major* infected skin was harvested and incubated with BFA for 5 hours. The samples were analyzed for the presence of P14 cells or OTI cells (D) and numbers of each were calculated (E). The OTI or P14 cells from the same infected ear of an LCMV immune *L. major* infected mouse were also incubated with BFA, monensin, and CD107a antibody then stained for additional surface and intracellular proteins (F). Data are representative of four independent experiments (n = 4–5 per group). Percentages are shown as mean ± SEM.

Given the increased number of CD8 T cells in the lesions of LCMV immune *L. major* infected mice, we hypothesized that this increase was due to an infiltration of LCMV specific CD8 T cells into the skin. To test this hypothesis we used two different approaches. First, we utilized a pool of 20 known LCMV peptides to restimulate cells taken from the infected ear, allowing us to broadly assess the LCMV responsiveness of the CD8 T cell population in the infected skin. Notably, a significant percentage of the CD8 T cells in the lesions were LCMV responsive, while there was a negligible response by cells from mice infected with *L. major* alone (Figure 5A and 5B). This result indicates that a large proportion of the CD8 T cells that have been recruited to the leishmanial lesion are LCMV specific. In addition, these results further demonstrate that although CD8 T cells did not appear to be making IFN-γ within the leishmanial lesions (Figure 4A), when stimulated with LCMV peptides they were capable of producing IFN-γ.

We next used a transgenic T cell transfer system to allow us to directly identify LCMV specific and *L. major* specific T cells (Figure 5C). We transferred CD45 congenic P14 T cells into B6 mice that were subsequently infected with LCMV or left uninfected. After thirty days, all mice received GFP^+^ OTI CD8 T cells (OVA specific) and were challenged with *L. major* expressing OVA (*L. major*-OVA). The course of infection, increased lesion size in LCMV immune mice, and parasite control were all unchanged by the presence of these transgenic T cells (Figure S2A and S2B). After *L. major*-OVA infection, OTI cells were detectable in the lesions of both groups of mice, although at a relatively low percentage of the total CD8 T cells (Figure 5D and 5E). This most likely reflects the fact that many of the CD8 T cells recognize leishmanial antigens other than OVA. Of the CD8 T cells in the lesions of LCMV immune *L. major* infected mice, half were P14 cells (Figure 5D and 5E), once again confirming that a large proportion of the CD8 T cells present were LCMV specific memory cells. Also, despite *L. major*-OVA infected mice receiving the same number of P14 cells as all other groups, there was no detectable expansion of P14 cells in mice infected with *L. major*-OVA alone (Figure 5D and 5E).

### LCMV specific cells express gzmB and markers of degranulation within leishmanial lesions

Using the transfer system described above, we found that neither P14 nor OTI cells from the lesions of LCMV immune *L. major* infected mice made much IFN-γ, suggesting that even leishmania-specific CD8 T cells may be making little IFN-γ in the lesions during a primary infection (Figure 5F). In contrast, when gzmB expression was evaluated, the majority of both the OTI and P14 cells were gzmB positive (Figure 5F). Additionally, when we analyzed CD107a expression, there were detectable CD107a positive P14 cells. While the frequency of CD107a positive OTI and P14 cells was not significantly different, the number of CD107a positive P14 cells was substantially higher than OTI cells and included staining that is clearly above background levels (Figure 5F). Interestingly, in the blood neither IFN-γ nor gzmB were expressed by OTI or P14 cells (Figure S2C), consistent with the idea that both antigen specific CD8 T cells and bystander CD8 T cells are induced to upregulate gzmB within the leishmanial lesions.

### No evidence for cross-reactivity between LCMV and *L. major*

In some studies, LCMV immune mice that are challenged with heterologous viruses have been shown to have overlapping or cross-reactive T cell responses [31]–[35]. One way to detect these cross-reactive T cell populations is restimulation with LCMV peptides. Therefore, to test for cross reactivity of LCMV and *L. major* we stimulated cells from the lesions of *L. major* infected mice with 20 different LCMV peptides. None of the LCMV peptides induced IFN-γ production in cells from *L. major* infected mice (Figure 5A and 5B). Since the hierarchy of immune dominant epitopes can be altered upon infection with a cross-reactive pathogen [33], we also examined three of the top immunodominant epitopes for LCMV using tetramer staining. We found no change in the frequency of the LCMV epitopes in LCMV immune mice following *L. major* infection in either the blood or the spleen (Figure S3A and S3B). Moreover, tetramer staining of the spleen and blood from naïve mice or mice infected with *L. major* alone revealed similar frequencies of tetramer positive cells, indicating that *L. major* infection alone did not expand these three epitopes (Figure S3A and S3B).

In an alternative approach to look for cross-reactivity, we sorted polyclonal CD44hi CD8 T cells from LCMV immune mice, CFSE labeled them and transferred these cells into three groups: one that was left naïve, one that was then infected with *L. major*, and one that was then infected with LCMV. After 7 days, the transferred LCMV memory cells were detectable in the spleen and LN of all three groups, but only in the skin of LCMV infected recipients and not in the *L. major* infected skin (Figure S3C). Analysis of CFSE dilution and number of recovered cells revealed that LCMV memory cells transferred into *L. major* infected recipients did not proliferate above the background levels seen in naïve mice (Figure S3D and S3E). As expected, LCMV memory cells transferred into mice that were subsequently infected with LCMV all proliferated and expanded significantly in number (Figure S3D and S3E). Finally, since heterologous infections can cause shifts in the memory repertoire that results in impaired protective responses to rechallenge [32], we tested the ability of LCMV immune mice infected with *L. major* to handle the virulent LCMV clone 13 virus, and found that *L. major* infection had not functionally altered the ability to mount an effective LCMV immune response (data not shown). Taken together, these experiments indicate that there is no evidence that a cross-reactive epitope is responsible for the activation of LCMV-specific CD8 T cells in *L. major* lesions.

### CD8 T cells are required for the increased pathology seen in LCMV immune mice infected with *L. major*

**Figure 6:**
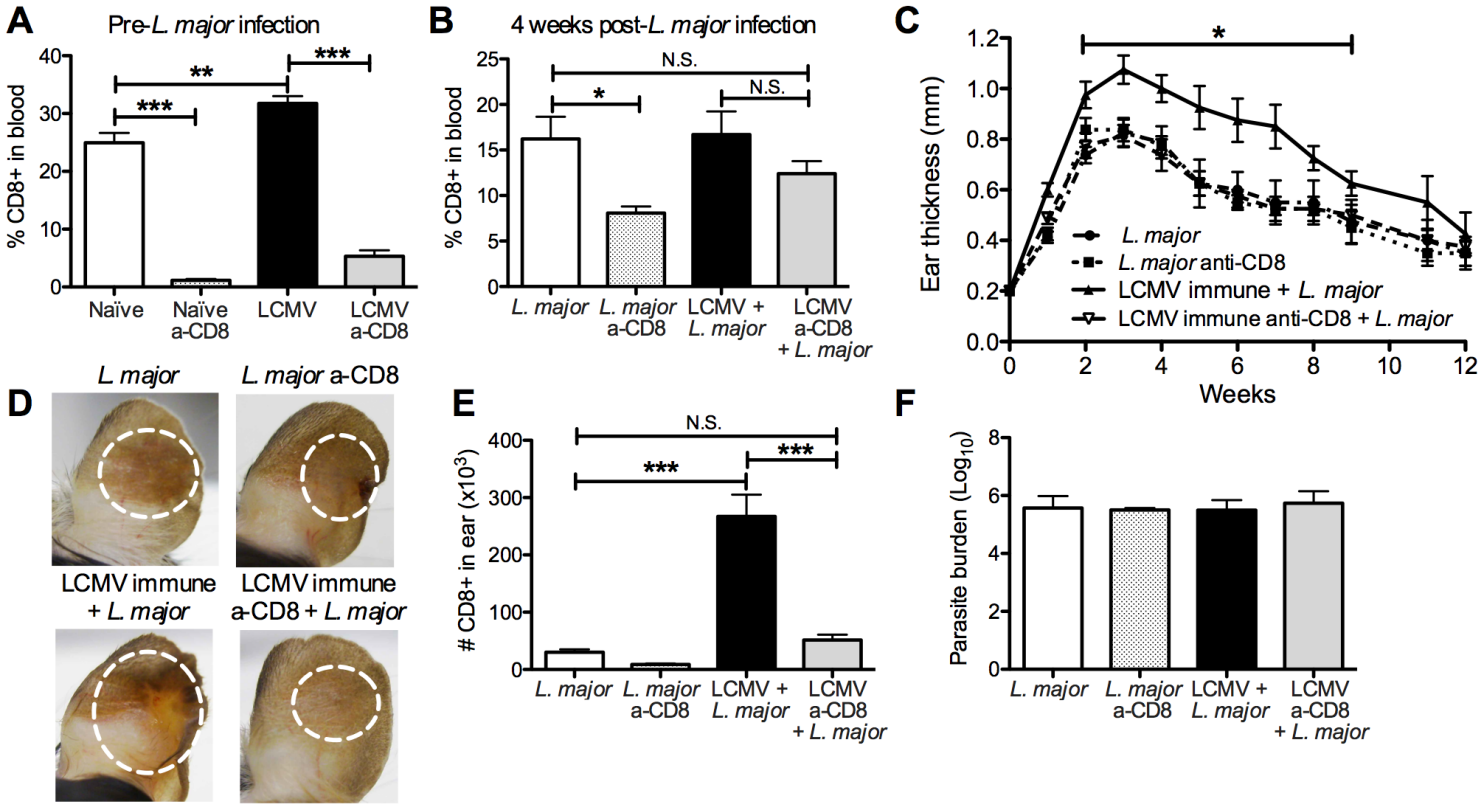
Exacerbated immunopathology is lost following depletion of CD8 T cells in LCMV immune mice prior to *L. major* infection. B6 mice were infected with LCMV or left uninfected for 45 days. Mice in each group were then treated with CD8-depleting antibody every 3 days for 15 days or left untreated. Blood was taken from animals in each group prior to *L. major* infection to assess CD8 depletion efficiency (A) and at 4 weeks post *L. major* infection to monitor reconstitution of the CD8 T cell compartment (B). All mice were with metacyclic *L. major* and ear thickness was measured weekly (C). Pictures were taken 4 weeks post *L. major* infection (D). Infected skin was taken at 4 weeks post infection and parasite burden was assessed using a limiting dilution assay (F). Infected skin was analyzed 4 weeks after *L. major* infection and the number of CD8 T cells present in each group was calculated (E). Data are representative of two independent experiments (n = 10 per group).

Given the significant, prolonged infiltration of LCMV specific memory CD8 T cells into leishmanial lesions, we wanted to determine if the exacerbated immunopathology was dependent on these CD8 T cells. To test this, we took naïve or LCMV immune mice and treated them with depleting anti-CD8 antibodies for 2 weeks. Although depletion of memory CD8 T cells was slightly less effective than that of naïve CD8 T cells, the treatment significantly decreased the percentage of circulating CD8 T cells in the blood of LCMV immune mice prior to *L. major* infection (Figure 6A). We then infected the CD8 depleted and control mice with *L. major* and monitored the course of infection. Prior to infection with *L. major*, we ceased treatment with anti-CD8 antibodies and at 4 weeks post *L. major* infection the circulating levels of CD8 T cells in LCMV immune CD8 depleted mice had recovered to levels comparable with *L. major* infected non-depleted mice (Figure 6B). Consistent with previous studies, under these conditions CD8 depletion did not influence the course of *L. major* infection [14], [36]–[38] (Figure 6C). As above, LCMV immune mice when challenged with *L. major* developed more severe lesions than control mice (Figure 6C). However, when CD8 depleted LCMV immune mice were infected with *L. major*, the lesion size and disease severity was similar to that seen in mice that had received *L. major* alone (Figure 6C and 6D). We found that the number of CD8 T cells in the lesions of LCMV immune CD8 depleted mice was similar to levels seen in mice that received *L. major* alone with no CD8 depletion, consistent with our ability to reduce the overall CD8 T cell population to normal levels (Figure 6E). In addition, the number of total cells infiltrating the LCMV immune CD8 depleted lesion was significantly reduced, reflecting a significant decrease in the number of monocytes and neutrophils present (data not shown). As observed above with LCMV immune mice, there was no change in the parasite burden in any of the groups (Figure 6F). Thus, these data clearly demonstrate that CD8 memory T cells are required for the increased immunopathology seen in LCMV immune mice.

### CD8 T cells induce immunopathology in an NKG2D dependent manner

**Figure 7:**
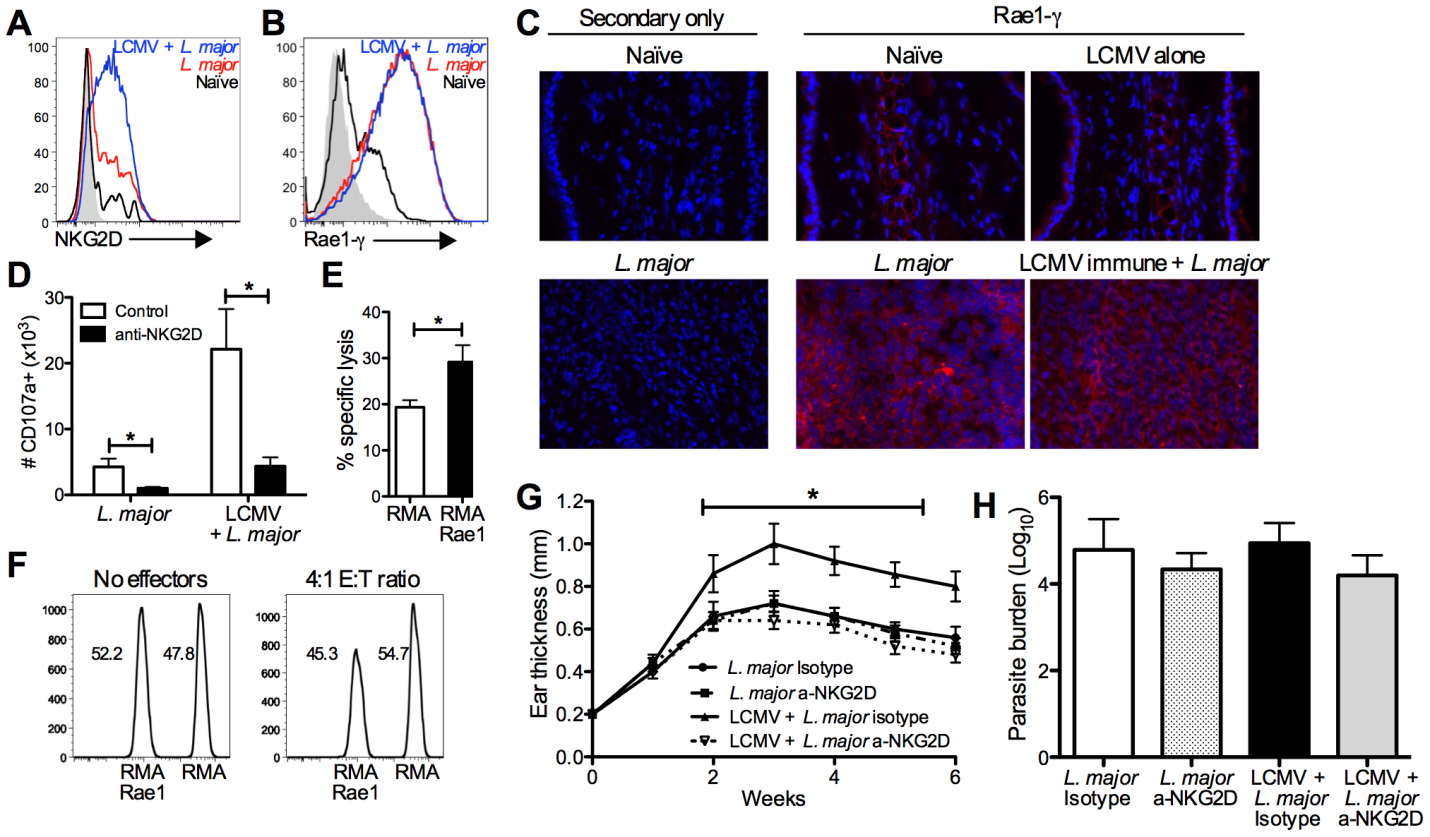
CD8 T cells induce immunopathology through engagement of NKG2D. B6 mice were infected with LCMV Armstrong or left uninfected. After 30 days, mice were infected with *L. major*. After 4 weeks, infected skin was taken for analysis by flow cytometry (A, B and D) or frozen for immunohistochemical staining (C). Cells were pregated on live, CD45+, CD8β+ (A and D) or live, CD45+, CD11b+ (B). Frozen sections were cut and stained with DAPI (blue) and anti-Rae1-γ (red) and imaged at 40× magnification (C). CD8 T cells from the lesions of LCMV immune *L. major* infected mice were harvested 5 weeks post infection and enriched by negative selection. RMA cells were labeled with a high dose of CTV and Rae1 expressing RMA cells were labeled with a low dose for use as target cells. Control and Rae1 expressing cells were mixed 1∶1 and incubated with LCMV immune CD8 T cells. Specific lysis was calculated for each group of target cells (E). The ratio of live target cells with or without effector cells is shown (F). Cells from the infected skin were divided and incubated with BFA, monensin, and CD107a antibody ±10 µg/ml of NKG2D blocking antibody and the number of CD107a+ cells calculated (D). B6 mice were infected with LCMV Armstrong or left uninfected. After 30 days, some mice in each group were treated with NKG2D blocking antibody or isotype control antibody. The following day mice were infected with *L. major*, and antibody treatment continued biweekly for the duration of the experiment. Ear thickness was measured weekly (G). Infected skin was taken 6 weeks post infection and parasite burden was assessed using an LDA (H). Data are representative of two independent experiments (n = 5 per group; A–D and G–H) or a single experiment (n = 5; E and F).

Having demonstrated that LCMV memory CD8 T cells are recruited and activated within leishmanial lesions, and that depletion of these cells abrogates increased disease, we further examined a mechanism by which the CD8 T cells were inducing immunopathology. In addition to expressing gzmB, LCMV memory CD8 T cells in leishmanial lesions of mice appeared to be degranulating, as measured by CD107a expression. This led us to examine alternate mechanisms for cytotoxicity by CD8 T cells that might be independent of cognate antigen interactions. A subset of memory CD8 T cells can express the activating NK cell receptor NKG2D. Engagement of this receptor can directly kill target cells, independent of TCR signals, through NKG2D recognition, as well as having a potent costimulatory effect on CD8 T cell activation [10], [39]–[42]. There are several families of ligands for NKG2D and these ligands are all stress induced, MHC class I homologs [43]. We found that CD8 T cells in the lesions of *L. major* infected mice expressed low levels of NKG2D, but CD8 T cells from LCMV immune *L. major* infected lesions had significantly higher expression of NKG2D (Figure 7A). This increased expression, combined with the significantly larger number of total CD8 T cells present in the LCMV immune *L. major* lesions represents a substantial increase in the number of NKG2D positive CD8 T cells in these lesions. NK cells from the lesions of both groups had comparable levels of expression of NKG2D (data not shown).

Having seen increased expression of NKG2D on CD8 T cells, we next examined the lesions for expression of NKG2D ligands, specifically a member of the Rae1 family of ligands, Rae1-γ. Analyzing the skin by flow cytometry revealed expression of Rae1-γ by CD11b positive cells at a very low level in naïve skin. However, after infection with *L. major* nearly all of the CD11b positive cells present in the lesion were expressing high levels of Rae1-γ (Figure 7B). Furthermore, analysis of Rae1-γ expression by immunohistochemistry demonstrated high levels of Rae1-γ within *L. major* lesions that are absent in naïve skin (Figure 7C). We observed expression of Rae1-γ both within the epidermis and widespread throughout the dermal layer of infected skin.

We next wanted to determine if we could link the levels of degranulation by CD8 T cells from the lesion to NKG2D engagement. To test this, we once again incubated cells from the lesions with anti-CD107a in the presence or absence of an antibody that blocks the interaction between NKG2D and its ligands. In the cultures where NKG2D interactions were blocked, there was significantly less CD107a staining in CD8 T cells from both groups (Figure 7D). These data indicate that NKG2D interactions contribute to the degranulation by CD8 T cells from the lesions.

NKG2D engagement can both be costimulatory or induce direct cytotoxicity in CD8 T cells [44]. To determine whether the NKG2D expressing CD8 T cells from the lesions of LCMV immune mice were capable of directly lysing NKG2D ligand expressing cells, we used the VITAL in vitro killing assay [45]. Our target cells were RMA cells that expressed Rae1 or control RMA cells that did not express any NKG2D ligands [5], [46]. We found that there was significantly more lysis of Rae1 expressing RMA cells than control RMA cells (Figure 7E). This could also be visualized by the selective loss of live Rae1 expressing RMA cells compared to control cells (Figure 7F). Taken together, these data suggest that CD8 T cells from the lesions of LCMV immune mice are capable of killing target cells in an NKG2D-dependent manner.

Ultimately we wanted to ascertain whether NKG2D expression by CD8 T cells was mediating the increased immunopathology observed in LCMV immune mice. We treated mice with NKG2D-blocking antibody or isotype control antibody starting one day before infection with *L. major* and twice a week after infection. We monitored lesion progression and found that while treatment with NKG2D-blocking antibody had no effect on the disease course in mice infected with *L. major* alone, it significantly reduced lesion size in LCMV immune mice infected with *L. major* (Figure 7G). As with CD8 depletion, despite completely reversing the increased immunopathology, there was no effect on parasite burden in any of the treatment groups (Figure 7H). Given that NKG2D is also an activating receptor on NK cells, we depleted NK cells during *L. major* infection and found that LCMV immune mice still exhibited exacerbated immunopathology (Figure S4). Taken together, these data demonstrate that LCMV memory CD8 T cells are recruited into leishmanial lesions, become activated, and induce immunopathology in an NKG2D dependent manner.

## Discussion

This study demonstrates that memory CD8 T cells induced by a previous infection can enhance the disease severity of a subsequent unrelated infection in the skin. Thus, challenge of LCMV or *Listeria* immune mice with *L. major* resulted in increased lesion size and exacerbated pathology without any change in parasite control. Analysis of the leishmanial lesions in LCMV immune mice showed a greater than four-fold increase in the number of CD8 T cells, at least half of which were LCMV specific. We demonstrated that LCMV memory T cells can migrate into leishmanial lesions, and because LCMV immune mice maintained an elevated frequency of activated CD8 T cells circulating in the blood long after the clearance of virus [30] we believe that the CD8 T cells in the lesions were from the blood. To test if these CD8 T cells were responsible for the increased pathology, LCMV immune mice were depleted of CD8 T cells prior to challenge with *L. major* and this depletion reversed the increased pathology observed in LCMV immune mice. Importantly, we further demonstrated that the CD8 T cells were meditating the increased pathology in an NKG2D dependent manner by blocking these interactions in vivo. Thus, our results show that bystander memory CD8 T cells can be recruited non-specifically to the skin during an infection-induced inflammatory response and instead of increasing protection, can exacerbate disease.

It has become increasingly clear that the study of a single pathogen in mice does not always reflect “real life” situations, where prevalent coinfections can have a significant impact on disease. For example, secondary bacterial infections are a common clinical complication during viral infections in the lung [47], [48]. Coinfection-induced changes in the host immune response can contribute to disease progression by reducing the ability to control the secondary pathogen itself or to control pathogen-induced tissue damage [49]–[51]. In addition to acute coinfections, chronic infections, such as helminths or mycobacteria, significantly impact the immune responses to other infections or vaccines (reviewed in [52]). In leishmaniasis, HIV coinfection contributes to enhanced disease, as does simultaneous infection with schistosomes [53], [54]. Taken together these studies argue that fully understanding the outcome of one infection may require knowledge about additional ongoing infections. Our study expands this concept to include the influence of previous infections. Thus, while it is clear that secondary infections occur and pose a significant health risk, every human has circulating memory T cells that could potentially impact the disease outcome for future infections. This makes understanding the role of bystander T cells in disease an important and distinct question for understanding human disease.

While it has been more than 20 years since bystander T cells were first hypothesized to participate in immune responses, their relative contribution to the immune response remains controversial [55]–[58]. In the context of viral infections, bystander T cell effects are often attributed to cross-reactivity and may therefore be TCR-driven [59]–[62]. While cross-reactive peptides have been identified for some virus pairs, it is a technically challenging endeavor and in many cases it is still unclear which epitopes are cross-reactive [31]–[35], [63], [64]. However, the recent use of TCR transgenic T cells has provided convincing results that memory CD8 T cells can be activated by inflammatory cytokines and contribute to immune responses without the need for cognate antigen stimulation [1], [2], [4], [6], [55], [65]. For example, protective immunity to *Listeria* can be generated using TCR transgenic memory cells even when the challenging strain of *Listeria* does not express the cognate antigen recognized by the transgenic T cells [2], [4]. IFN-γ production and proliferation of memory cells are two major readouts that have been used to demonstrate cross-reactivity [34], [59], [60], [66], and LCMV memory cells did not produce IFN-γ in response to restimulation with leishmanial antigen and did not proliferate in response to infection with *L. major* (Figure 2F and S3). Changes in epitope dominance are also an indication that cross-reactivity is playing role [33], however there was no change in the dominance hierarchy of major LCMV epitopes following *L. major* infection and no expansion of these epitopes was observed in animals infected with *L. major* alone (Figure S3A and S3B). Moreover, despite *L. major* infected animals receiving the same number of P14 transgenic T cells prior to infection as the LCMV immune group, there was no expansion or activation of these cells in response to *L. major* infection alone. Yet a majority of the P14s present within the lesions of LCMV immune *L. major* infected mice were activated and expressing gzmB (Figure 5D and 5E). Additionally, we observed the same phenotype and activation of CD8 T cells from *Listeria* immune animals during *L. major* infection (Figure 2 and data not shown). Overall, while the complexity of these pathogens leaves open the possibility of some undetected cross-reactivity being involved in the responses we have observed, all of our results are consistent with activation of bystander CD8 T cells due to the inflammatory milieu present in the lesion.

A key difference between many of the previous studies of bystander T cell responses and our current findings is that instead of increased protection, we find that bystander memory CD8 T cells have no impact on parasite control, but instead increase pathology. As most studies have monitored the response of bystander T cells during acute infections [2], [4], [59], [60], it is possible that activation of bystander T cells in a chronic, inflammatory environment is responsible for these differential effects. The sustained recruitment of cells over many weeks in leishmaniasis may lead to a greater accumulation of bystander CD8 T cells that can promote pathology. Our study is also the first to characterize the role of bystander CD8 T cells in the skin, where the pathologic effects of the bystander CD8 T cell activation can be directly visualized. The most striking difference between our results and prior studies is that following *L. major* infection the bystander CD8 T cells recruited to the skin made gzmB, but little IFN-γ. This is in contrast to other infections where bystander CD8 T cells were activated and quickly produced IFN-γ, to provide increased protection [4], [59], [60], [62]. What accounts for this difference in effector function is unclear, however it may be that the inflammatory response induced by *L. major* is insufficient to promote IFN-γ production. Different cytokine environments can differentially regulate the effector profile of CD8 T cells, with cytokines like IL-12 and IL-18 inducing high amounts of IFN-γ, while IL-15 induces the upregulation of gzmB [1], [4], [6], [67], [68]. We are currently examining the role of these cytokines on CD8 T cell activation within leishmanial lesions. Overall, our work suggests that the nature of the inflammation, including duration, location, and composition, will impact whether bystander CD8 T cell activation will be protective or pathologic.

CD8 T cells have many effector functions that could be playing a role in pathogenicity. For example, CD8 T cell derived CCL3 and CCL4 increases recruitment and activation of the inflammatory cells into tissues [69]. Production of chemokines by tissue resident memory CD8 T cells is also important for recruitment of CD8 T cells to sites of inflammation [70]. Similarly, CD8 T cells produce inflammatory cytokines, such as IFN-γ, TNF-α, and IL-17 that can promote tissue destruction [71]–[73]. However, the levels of these particular cytokines were low or absent in CD8 T cells within the leishmanial lesions, making them unlikely to be playing a major role (Figures 4 and 5 and data not shown). Additionally, large changes in IFN-γ production would most likely result in increased parasite killing, which was not observed (Figure 2E). While bystander CD8 T cells would not be expected to exhibit antigen-specific cytolytic activity, we have demonstrated that LCMV specific CD8 T cells can act non-specifically through an NKG2D-dependent mechanism (Figure 7). Recent work has shown that NKG2D engagement on bystander CD8 memory cells can serve as an innate-like killing mechanism for early control of bacterial burdens in *Listeria* infection [5]. Here we highlight the pathologic role this pathway can play, leading to excessive cell death and the induction of enhanced inflammation. These experiments emphasize an underappreciated effector function of CD8 T cells that can have serious consequences for disease progression in leishmaniasis, and potentially other diseases as well.

The formation of a protective memory T cell pool is a crucial part of an effective immune response. However, unlike highly controlled mouse models of disease, humans encounter many pathogens throughout their life that can increase the size and diversity of their immunological memory pool. Determining how this pool of memory T cells impacts future immune responses to unrelated pathogens may be critical for understanding the diversity observed in human disease. While differences in each individual's response to a given infection is related to pathogen dose and the genetic and physiological status of the host, our work suggests that the immunological history of a patient may also play a role. Leishmania infections have a very broad spectrum of clinical disease ranging from subclinical infection to ulcerating skin lesions to destruction of the mucosal tissue [15]–[17]. Both parasite and host factors have been implicated in this diversity, but our current work indicates that the immunological history of the patient may also directly impact the severity of leishmanial disease. In support of this idea, non-leishmania specific CD8 T cells are found within human leishmanial lesions [74] and there is a correlation between the number of intralesional CD8 T cells and disease severity [24], [25]. Taken together, our studies demonstrate that bystander CD8 T cell activation can play a role in leishmanial lesion progression through the engagement of an NK cell receptor, NKG2D. On a broader level, our studies highlight the potential importance of heterologous immunological responses, which remains an under-appreciated aspect of the host immune response to infection. Understanding how prior immune responses influence subsequent infections and infection-induced pathology will be important for designing new and effective therapeutic treatments. This work has identified NKG2D and its ligands as potential therapeutic targets in leishmaniasis and has broad implications for potential role of this pathway in other infections where excessive tissue damage and immunopathology are observed.

## Materials and Methods

### Animals

Female C57BL/6 mice and B6-Ly5.2/Cr (CD45.1) (6 weeks old) were purchased from the National Cancer Institute (Fredericksburg, MD). RAG2/OT-I mice were purchased from Taconic Farms. Mice expressing eGFP in all T cells were originally obtained from Ulrich van Andrian (Harvard University) and crossed with RAG2/OTI mice to generate GFP+ OTI CD8 T cells. CD45.1+ P14 mice bearing the H-2D^b^ gp33-specific T cell receptor were maintained in our animal colony [75]. Animals were housed in a specific pathogen-free environment and tested negative for pathogens in routine screening. This study was carried out in strict accordance with the recommendations in the Guide for the Care and Use of Laboratory Animals of the National Institutes of Health. The protocol was approved by the Institutional Animal Care and Use Committee, University of Pennsylvania Animal Welfare Assurance Number A3079-01.

### Parasite, virus, and bacterial infections

*L. major* parasites (Friedlin) or *L. major* expressing ovalbumin (*L. major*-OVA) were grown to the stationary phase in Schneider's Drosophila medium (Gibco) supplemented with 20% heat-inactivated FBS (Gibco) and 2 mM L-glutamine (Sigma) at 26°C [76]. Metacyclic promastigotes were isolated from 4–5 day old stationary cultures by density gradients [77]. Mice were infected with 2×10^6^ metacyclic parasites injected intradermally into the ear. Lesion development was monitored weekly by taking measurements of ear thickness with digital calipers (Fisher Scientific). Parasite burden in lesion tissues was assessed using a limiting dilution assay as previously described [78]. For viral infections, mice were infected with 2×10^5^ PFU of LCMV Armstrong strain by i.p. injection. For bacterial infections, mice were prime-boosted with an initial infection of 10^3^ *Listeria monocytogenes* expressing ovalbumin (*Listeria*-OVA) followed 30 days later by an additional boost infection of 5×10^4^ *Listeria*-OVA, both doses given intravenously.

### T cell transfers

P14 chimeras were made by transferring 2×10^4^ congenically marked P14 CD8 T cells 1 day prior to infection with LCMV. Prior to transfer of effector or memory P14 CD8 T cells from P14 chimeras, splenocytes were enriched for T cells using the Pan T cell Isolation Kit II according to the manufacturer's instructions (Miltenyi Biotec). Enriched fractions were analyzed by flow cytometry and 5×10^5^ P14 CD8 T cells were transferred. Naïve P14 CD8 T cells were not enriched prior to transfer. When OTI CD8 T cells were used, 2×10^5^ GFP+ cells were transferred 1 day prior to *L. major*-OVA infection.

### Histology

*L. major* infected ears were taken at the peak of lesion formation, fixed in 10% buffered formalin, and embedded in paraffin. Longitudinal 5 µm sections were cut and stained with hematoxylin and eosin or Verhoeff's stain. For analysis of Rae1-γ, skin was taken at the peak of lesion formation and frozen in Tissue-Tek OCT compound (Electron Microscopy Sciences). Longitudinal 5 µm sections were cut and stained with biotinylated anti-mouse Rae1-γ (eBioscience, CX1). Sections were blocked prior to staining with Avidin Biotin blocking solution according to the manufacturer's instructions (Thermo Scientific). Photographs were taken with a Nikon Digital Sight DS-Fi1 Color system, (Nikon eclipse E600 Microscope).

### Flow cytometry

For flow cytometry, cells were isolated from ears, draining lymph nodes, spleens or peripheral blood. For ears, dermal sheets were separated and incubated in incomplete IMDM+GlutaMAX (Gibco) containing 0.25 µg/mL of Liberase TL (Roche, Diagnostics Corp.) and 10 µg/mL DNase I (Sigma-Aldrich) for 90 minutes at 37°C. Ears, draining lymph nodes, and spleens were mechanically dissociated by smashing through a 40-µm cell strainer (Falcon) in PBS containing 0.05% BSA and 20 µM EDTA. Splenocytes were incubated for <1 minute with ACK lysing buffer (Lonza) to lyse red blood cells. When indicated, cells were incubated at 4×10^6^ cells/ml with Brefeldin A (BFA, 3 µg/ml final concentration) (eBiosciences) alone for 5 hours, with a pool of 20 LCMV peptides (each peptide at a final concentration of 0.4 µg/ml) and BFA for 6 hours, or with phorbol myristate acetate (PMA) (Sigma, 100 ng/ml final concentration) and ionomycin (Sigma, 1 µg/ml final concentration) and BFA for 4–6 hours before staining for flow cytometry. In experiments analyzing CD107a expression, cells were incubated with BFA, monensin (eBioscience, 2 µM final concentration), and anti-CD107a (eBioscience) for 6 hours. Fixable Aqua dye (Invitrogen) was added to assess cell viability. Cells were then incubated with FC block (anti-CD16/32, heat inactivated mouse sera and Rat IgG) followed by fluorochrome-conjugated antibodies for surface markers CD45, CD45.1, CD45.2, CD8β, CD4, CD44, CD62L, CD11b, Ly6C, and/or Ly6G (1A8) (all eBioscience) and were fixed with 2% paraformaldehyde (Electron Microscopy Sciences). For intracellular staining, cells were previously permeabilized with 0.2% of saponin buffer and stained for IFN-γ, gzmB, TNF-α, and/or IL-17A (eBioscience or Invitrogen). The data were collected using an LSR Fortessa flow cytometer (BD Bioscience) and analyzed with FlowJo software (Tree Star). For sorting LCMV memory cells, splenocytes were stained for surface markers (CD4, CD8β and CD44) and CD8β^+^ CD44^hi^ cells were sorted on a FACSAria II (BD Biosciences).

### Histopaque

Peripheral blood was collected into a 4% sodium citrate solution. White blood cells were isolated by underlaying with Histopaque-1083 (Sigma) and spinning for 20 minutes at 400×g at room temperature.

### Ear homogenization

Whole ears were cut into <1 mm pieces and placed in ice cold PBS with a protease inhibitor cocktail (Sigma). Samples were homogenized using the FastPrep-24 (MP Biomedicals) and spun for 20 minutes at 13000 rpm at 4°C in a microcentrifuge. The supernatants were removed and stored at −80°C until analysis by ELISA as described below.

### Leishmanial antigen restimulation and ELISAs

Leishmanial antigen was obtained from stationary-phase promastigotes of *L. major* by resuspending parasites at 1×10^9^ parasites/ml in PBS and conducting 20 freeze/thaw cycles. For measurements of antigen-specific cytokine production, the infected skin draining retroauricular lymph node was removed, mechanically dissociated, and single cell suspensions were prepared. Cells were resuspended in complete IMDM+GlutaMAX (Gibco) supplemented with 10% heat inactivated FBS (Gibco), 2 mM l-glutamine (Sigma), 100 U of penicillin and 100 µg of streptomycin (Sigma) per mL and 0.05 µM of β-mercaptoethanol (Sigma). Cells were plated at 4×10^6^ cells/mL in 1 ml in 48-well plates. Cells were incubated at 37°C in 5% CO_2_ with 20×10^6^ *L. major* parasites/mL. Supernatants were collected after 72 hours and stored at −20°C until they were assayed by sandwich ELISA using paired monoclonal antibody to detect IFN-γ, IL-4 or IL-17 (eBioscience). Cytokine concentrations were calculated from standard curves with detection limit of 0.03 ng/mL for IFN-γ, 0.015 ng/mL for IL-17A and 7 Units/mL of IL-4. Granzyme B was analyzed by ELISA using a mouse granzyme B Duoset kit (R&D Systems).

### VITAL assay

A VITAL assay was performed and analyzed as described [45]. Briefly, CD45.1+ CD8 T cells for the VITAL assay were enriched from the lesions of LCMV immune *L. major* infected mice at 4 weeks post *L. major* infection using negative selection kit for CD8 T cells according to the manufacturer's instructions (Miltenyi Biotec). Target cells for this assay were control RMA cells that do not express any NKG2D ligands or RMA cells that had been stably transfected with Rae1, originally made by L. Lanier [46]. RMA control cells were labeled with a high dose (1 µM) of CTV (Cell trace violet) and Rae1 expressing RMA cells were labeled with a low dose (0.02 µM) of CTV for use as target cells according to the manufacturer's instructions (Life Technologies). Control and Rae1 expressing cells were mixed 1∶1 and incubated with LCMV immune CD8 T cells from the lesion at an effector to target ratio of 4∶1 for 5 hours in vitro. The CTV low-to-high ratio of live cells was determined by analysis on an LSR Fortessa flow cytometer. Dead cells were excluded by propidium iodide staining. Expression of Rae1 was confirmed using a pan-Rae1 antibody (R&D systems).

### In vivo antibody treatment

For CD8 depletion, C57BL/6 mice were either infected with LCMV or left uninfected for 45 days. Both groups were then treated with 250 µg anti-CD8 (Clone 53-6.72; BioXCell) in PBS every 3 days for 15 days for a total of 5 doses. On the final day of antibody treatment, all groups were infected with *L. major* as described above. NK1.1 depleting (500 µg/dose; Clone PK136; BioXCell), NKG2D blocking (200 µg/dose; Clone HMG2D; BioXCell), and Hamster IgG control (200 µg/dose; BioXCell) antibodies were given 1 day prior to infection with *L. major* and twice weekly for the duration of the experiment.

### Statistics

Results represent means ± SEM. Data were analyzed using Prism 5.0 (GraphPad Software, San Diego, CA). Statistical significance was determined using unpaired, one-tailed Student's *t* test with p values given as: *p<0.05; **p<0.001; and ***p<0.0001; ns p>0.05. Results with a p value≤0.05 were considered significant.

## Supporting Information

Figure S1 **CD8 T cell population is significantly larger in LCMV immune mice while CD4 populations remain similar.** B6 mice were infected with LCMV or left uninfected. After 30 days, mice were infected with metacyclic *L. major*. After 4 weeks, blood was taken from both groups and white blood cells were isolated. Cells were stained for surface T cell and activation markers. The proportion of CD4 T cells (A) or CD8 T cells (B) that were naïve (CD44^neg^ CD62L^hi^), central memory (CD44^hi^CD62L^hi^), or effector memory (CD44^hi^CD62L^lo^) was calculated as a frequency of cells in the lymphocyte gate. Data are representative of four independent experiments (n = 5 per group).
(TIFF)

Figure S2 **Transfer of P14 CD8 T cells and OTI T cells does not alter lesion progression or parasite control.** P14 cells were transferred into B6 mice and the next day a group was infected with LCMV. After 30 days, GFP+ OTI cells were transferred and all mice were infected with *L. major*-OVA. Ear thickness was measured weekly (A). Infected skin was taken at various time points post infection and parasite burden was assessed using a limiting dilution assay (B). After 4 weeks, blood was taken from both groups and white blood cells were isolated. Cells were stained for surface and intracellular proteins (C). Data are representative of two independent experiments (n = 4–5 per group). Percentages are shown as mean ± SEM.
(TIFF)

Figure S3 **No evidence for cross-reactivity between LCMV and** ***L. major*** **.** B6 mice were infected with LCMV Armstrong or left uninfected. After 30 days, mice were infected with *L. major*. After 4 weeks, blood (A) and spleens (B) were taken for analysis by flow cytometry and stained with three different tetramers and other surface markers. B6 mice were infected with LCMV. After 30 days, splenocytes were harvested and CD44^hi^ CD8 T cells were sorted. Equal numbers of CD44^hi^ CD8 T cells were CFSE labeled and transferred into mice that were then left uninfected, infected with *L. major*, or infected with LCMV. After 1 week, spleens, skin and draining lymph nodes were harvested and transferred CD45.2+ cells were analyzed by flow cytometry. Representative plots (C and D) and pooled data (E) are shown. Data are representative of a single experiment (n = 2–3 mice per group).
(TIFF)

Figure S4 **NK cell are not required for increased immunopathology in LCMV immune mice.** B6 mice were infected with LCMV Armstrong or left uninfected. After 30 days, some mice in each group were treated with NK1.1 depleting antibody or isotype control antibody. The following day all mice were infected with *L. major*, and antibody treatment continued twice weekly for the duration of the experiment. Infected skin was taken at 6 weeks post infection and depletion of NK cells was assessed by flow cytometry (A). Ear thickness was measured weekly (B). Infected skin was taken 6 weeks post infection and parasite burden was assessed using an LDA (C). Data are representative of a single experiment (n = 5 per group).
(TIFF)
