# Supplementary figures and images for: Engagement of NKG2D on Bystander Memory CD8 T Cells Promotes Increased Immunopathology following Leishmania major Infection
Source: PLoS Pathog. 2014 Feb 27;10(2):e1003970. doi: 10.1371/journal.ppat.1003970 (PMC3937277; doi:10.1371/journal.ppat.1003970)

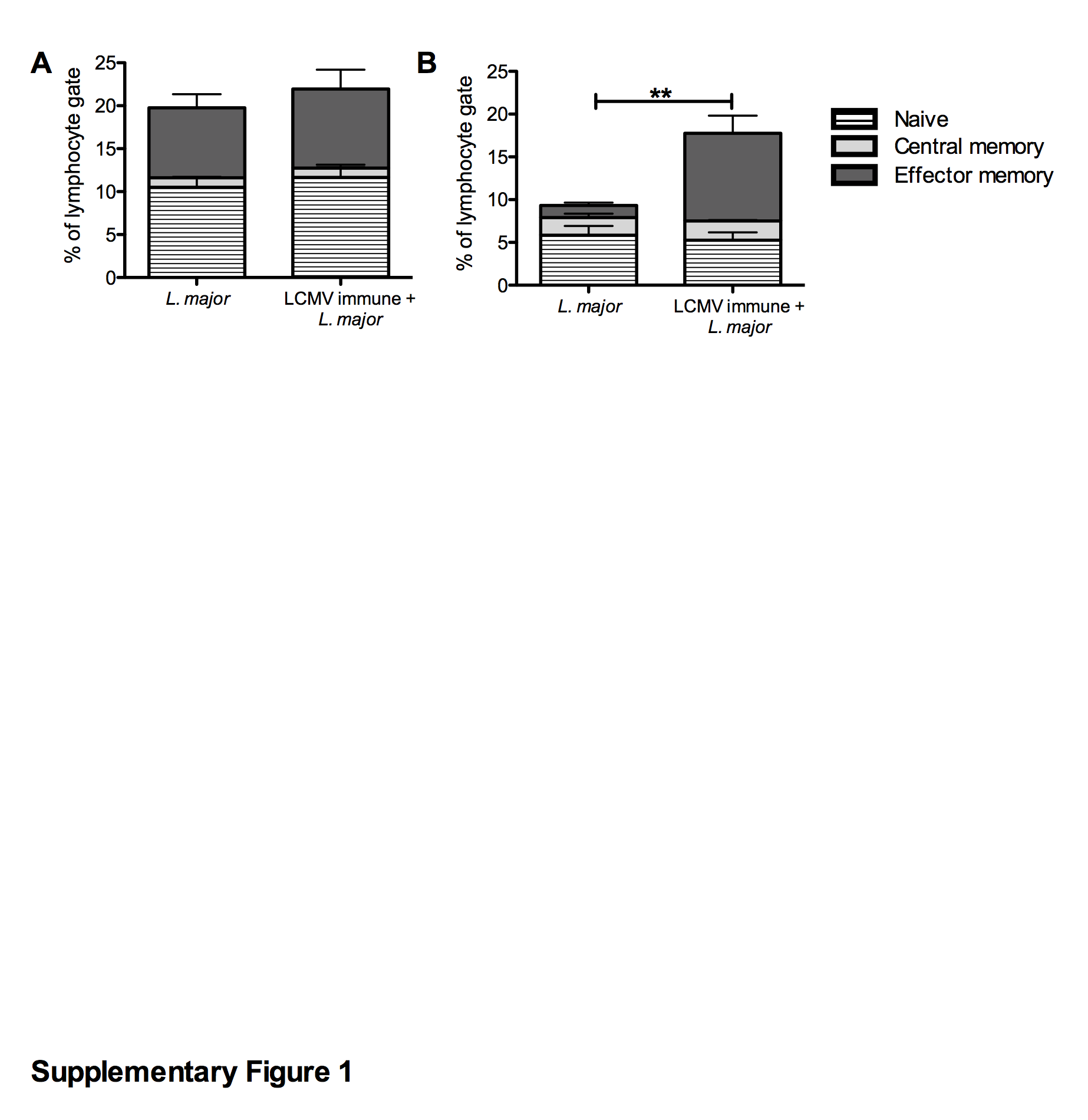

Supplement: Figure S1 — CD8 T cell population is significantly larger in LCMV immune mice while CD4 populations remain similar. B6 mice were infected with LCMV or left uninfected. After 30 days, mice were infected with metacyclic L. major. After 4 weeks, blood was taken from both groups and white blood cells were isolated. Cells were stained for surface T cell and activation markers. The proportion of CD4 T cells (A) or CD8 T cells (B) that were naïve (CD44neg CD62Lhi), central memory (CD44hiCD62Lhi), or effector memory (CD44hiCD62Llo) was calculated as a frequency of cells in the lymphocyte gate. Data are representative of four independent experiments (n = 5 per group). (TIFF) [file ppat.1003970.s001.tiff]

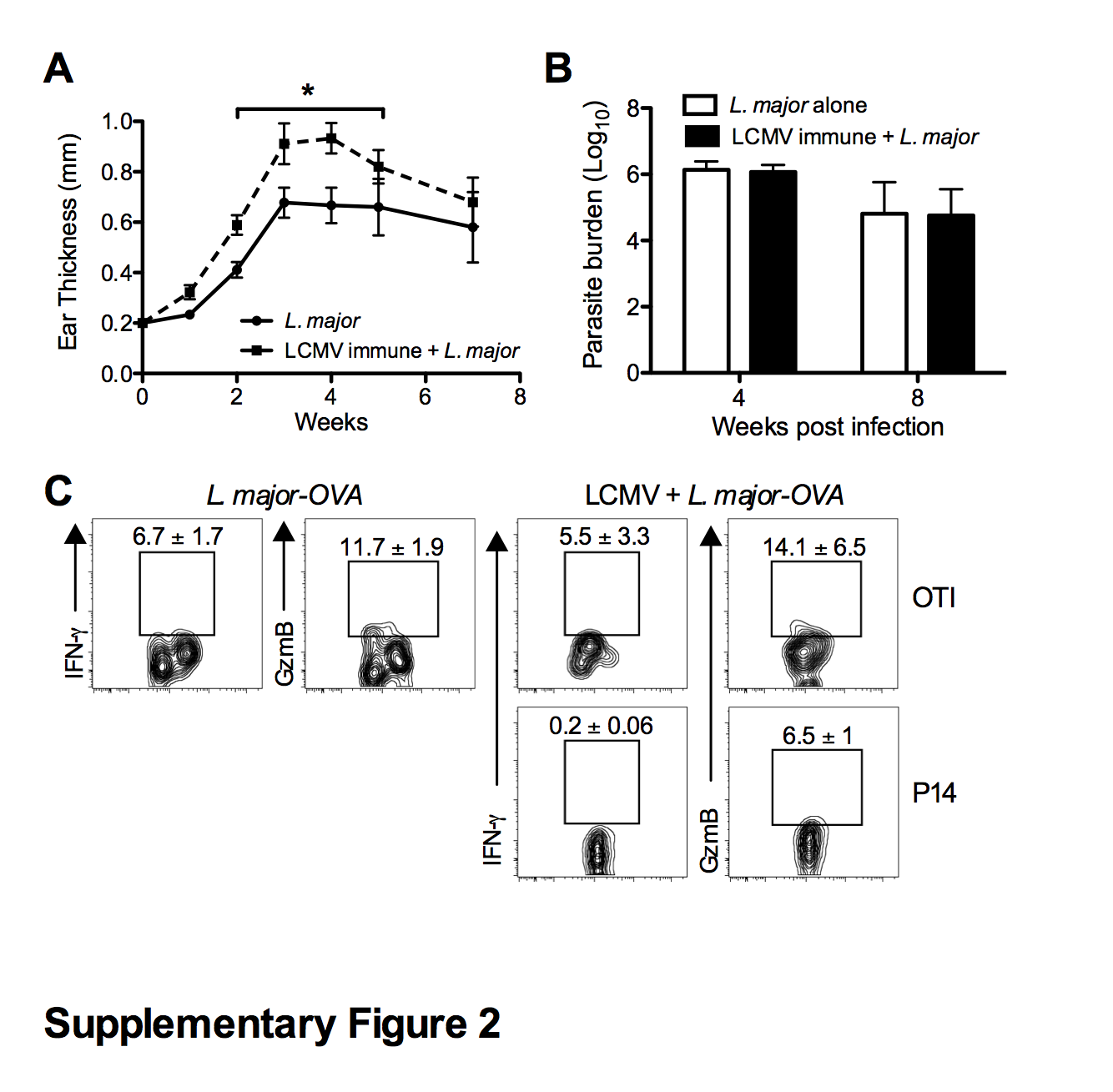

Supplement: Figure S2 — Transfer of P14 CD8 T cells and OTI T cells does not alter lesion progression or parasite control. P14 cells were transferred into B6 mice and the next day a group was infected with LCMV. After 30 days, GFP+ OTI cells were transferred and all mice were infected with L. major-OVA. Ear thickness was measured weekly (A). Infected skin was taken at various time points post infection and parasite burden was assessed using a limiting dilution assay (B). After 4 weeks, blood was taken from both groups and white blood cells were isolated. Cells were stained for surface and intracellular proteins (C). Data are representative of two independent experiments (n = 4–5 per group). Percentages are shown as mean ± SEM. (TIFF) [file ppat.1003970.s002.tiff]

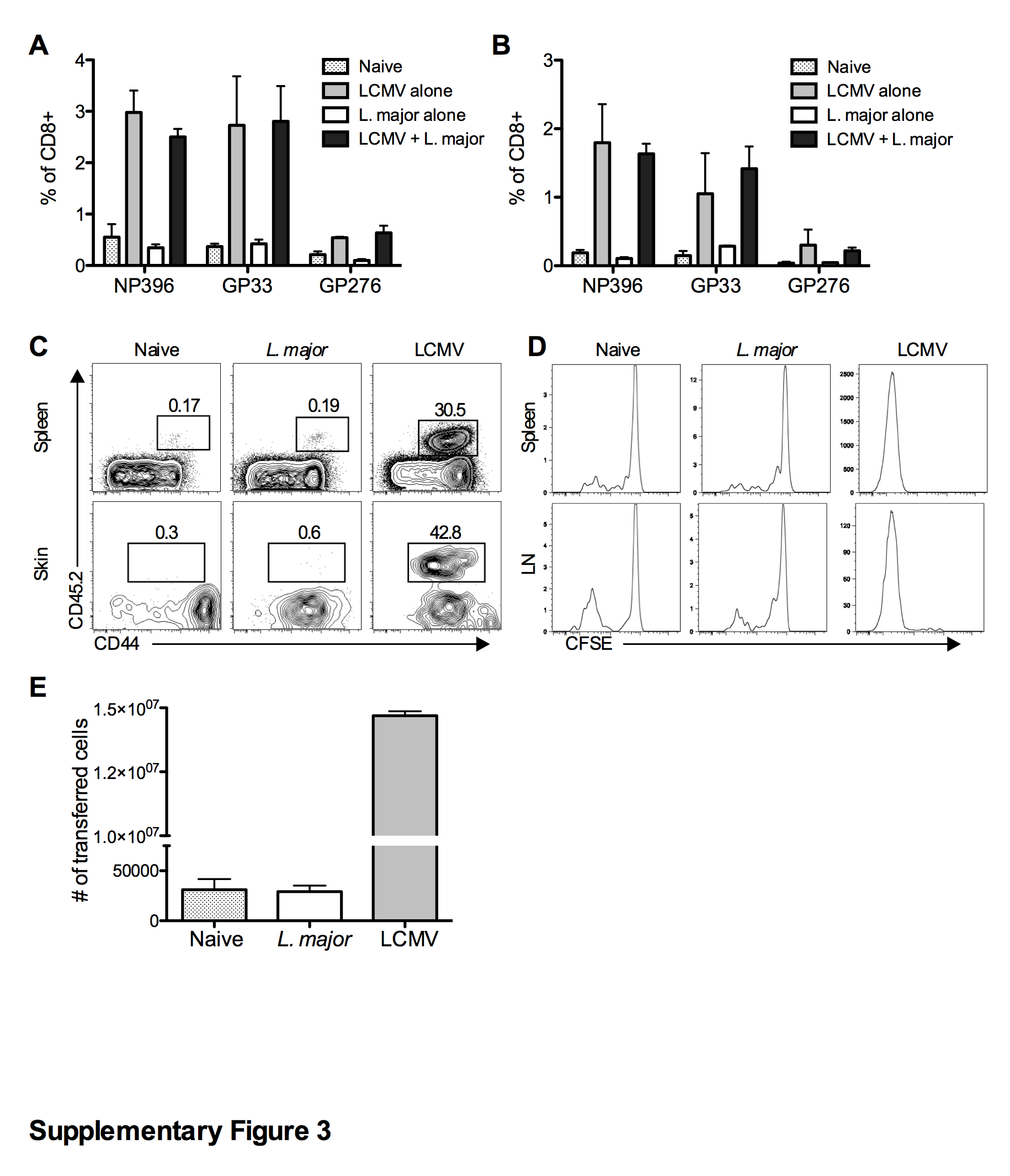

Supplement: Figure S3 — No evidence for cross-reactivity between LCMV and L. major . B6 mice were infected with LCMV Armstrong or left uninfected. After 30 days, mice were infected with L. major. After 4 weeks, blood (A) and spleens (B) were taken for analysis by flow cytometry and stained with three different tetramers and other surface markers. B6 mice were infected with LCMV. After 30 days, splenocytes were harvested and CD44hi CD8 T cells were sorted. Equal numbers of CD44hi CD8 T cells were CFSE labeled and transferred into mice that were then left uninfected, infected with L. major, or infected with LCMV. After 1 week, spleens, skin and draining lymph nodes were harvested and transferred CD45.2+ cells were analyzed by flow cytometry. Representative plots (C and D) and pooled data (E) are shown. Data are representative of a single experiment (n = 2–3 mice per group). (TIFF) [file ppat.1003970.s003.tiff]

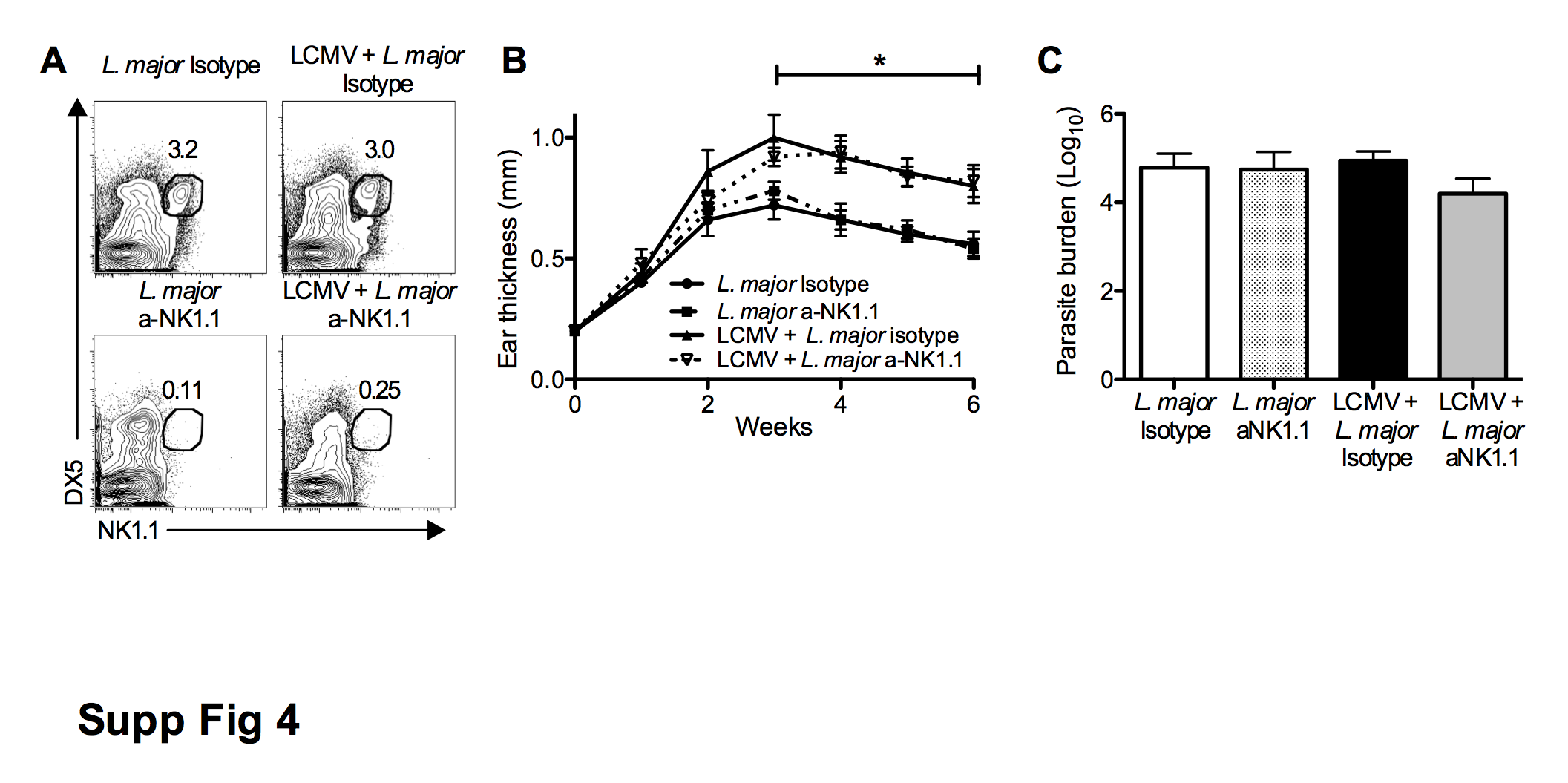

Supplement: Figure S4 — NK cell are not required for increased immunopathology in LCMV immune mice. B6 mice were infected with LCMV Armstrong or left uninfected. After 30 days, some mice in each group were treated with NK1.1 depleting antibody or isotype control antibody. The following day all mice were infected with L. major, and antibody treatment continued twice weekly for the duration of the experiment. Infected skin was taken at 6 weeks post infection and depletion of NK cells was assessed by flow cytometry (A). Ear thickness was measured weekly (B). Infected skin was taken 6 weeks post infection and parasite burden was assessed using an LDA (C). Data are representative of a single experiment (n = 5 per group). (TIFF) [file ppat.1003970.s004.tiff]
